# The effectiveness of a Housing First adaptation for ethnic minority groups: findings of a pragmatic randomized controlled trial

**DOI:** 10.1186/s12889-016-3768-4

**Published:** 2016-10-21

**Authors:** Vicky Stergiopoulos, Agnes Gozdzik, Vachan Misir, Anna Skosireva, Aseefa Sarang, Jo Connelly, Adam Whisler, Kwame McKenzie

**Affiliations:** 1 Centre for Addiction and Mental Health, Toronto, ON Canada; 2 Centre for Urban Health Solutions, St. Michael’s Hospital, Toronto, ON Canada; 3 Department of Psychiatry, University of Toronto, Toronto, ON Canada; 4 Across Boundaries, Toronto, ON Canada; 5 Inner City Family Health Team, Toronto, ON Canada; 6 Health Equity Office, Centre for Addiction and Mental Health, room 2010, 33 Russell Street, Toronto, ON M5S 2G8 Canada

**Keywords:** Homelessness, Mental illness ethnic minority, Housing

## Abstract

**Background:**

Little is known about the effectiveness of Housing First (HF) among ethnic minority groups, despite its growing popularity for homeless adults experiencing mental illness. This randomized controlled trial tests the effectiveness of a HF program using rent supplements and intensive case management, enhanced by anti-racism and anti-oppression practices for homeless adults with mental illness from diverse ethnic minority backgrounds.

**Methods:**

This unblinded pragmatic field trial was carried out in community settings in Toronto, Canada. Participants were 237 adults from ethnic minority groups experiencing mental illness and homelessness, who met study criteria for moderate needs for mental health services. Participants were randomized to either adapted HF (*n* = 135) or usual care (*n* = 102) and followed every 3 months for 24 months. The primary study outcome was housing stability; secondary outcomes included physical and mental health, social functioning, quality of life, arrests and health service use. Intention to treat statistical analyses examined the effectiveness of the intervention compared to usual care.

**Results:**

During the 24-month study period, HF participants were stably housed a significantly greater proportion of time compared to usual care participants, 75 % (95 % CI 70 to 81) vs. 41 % (95 % CI 35 to 48), respectively, for a difference of 34 %, 95 % CI 25 to 43. HF also led to improvements in community integration over the course of the study: the change in the mean difference between treatment groups from baseline to 24-months was significantly greater among HF participants compared to those in usual care (change in mean difference = 2.2, 95 % CI 0.06 to 4.3). Baseline diagnosis of psychosis was associated with reduced likelihood of being housed ≥ 50 % of the study period (OR = 0.37, 95 % CI 0.18 to 0.72).

**Conclusion:**

Housing First enhanced with anti-racism and anti-oppression practices can improve housing stability and community functioning among ethnically diverse homeless adults with mental illness.

**Trial registration:**

International Standard Randomized Control Trial Number Register Identifier: ISRCTN42520374, assigned August 18, 2009.

**Electronic supplementary material:**

The online version of this article (doi:10.1186/s12889-016-3768-4) contains supplementary material, which is available to authorized users.

## Background

Ethnic differences in access to, use and outcomes of mental health care have been reported in the US, UK and Canada [1–3]. The literature on health disparities faced by individuals from ethnic minority groups experiencing both homelessness and mental illness, or how to address them, however, is scant [4, 5]. Homeless individuals experience high rates of mental illness, often in conjunction with substance misuse [6]; this population experiences poor access to care, increased morbidity and premature mortality [6], with ethnic disparities possibly contributing to additional disadvantage. Housing First (HF) and similar consumer-driven programs have emerged as effective interventions for homeless adults experiencing mental illness, offering participants immediate access to independent housing and mental health support services [7, 8], without the strict requirements of “housing readiness” (sobriety and acceptance of psychiatric treatment) imposed by more traditional “treatment first” interventions [9]. The At Home/Chez Soi study was a randomized field trial of HF conducted between 2009 and 2013 in five sites across Canada to assess the effectiveness of HF with either assertive community treatment or intensive case management for homeless adults with mental illness in diverse service delivery contexts [10]. In Toronto, Canada’s largest and most ethnically diverse urban centre, in addition to testing the effectiveness of HF for a representative sample of homeless adults with mental illness, an additional trial examined the effectiveness of a HF adaptation among ethnic minority groups with moderate needs for mental health support services. In consultation with local community agencies, an adapted HF intervention was developed which incorporated anti-racist and anti-oppressive practices with the aim of improving outcomes for black and ethnic minority participants by recognizing and addressing the impact of racism and oppression on their lives [11]. This paper examines the effectiveness of the adapted HF intervention, compared to usual care, in keeping homeless people from black and minority ethnic groups in stable housing and in improving their social functioning, quality of life and physical and mental health.

## Methods

### Study design and setting

We conducted a two-year randomized controlled trial in Toronto, part of the At Home/Chez Soi cross-site project, in order to assess the effectiveness of HF with intensive case management for homeless adults with mental illness from diverse ethnic minority groups. The protocol for the overall At Home/Chez Soi project [10] and the Toronto site specifically [12] have been described previously. Prior to randomization, all participants were first stratified either to a high needs or moderate needs group based on their level of need for mental health services. At the Toronto site, participants in the moderate need group were further stratified by ethnicity (belonging to ethnic minority groups or not). This article focuses exclusively on study outcomes among the sample of moderate needs participants who belong to an ethnic minority group.

The study was approved by the Research Ethics Board (#09-208) of St. Michael’s Hospital in Toronto, and was registered with the International Standard Randomized Control Trial Number (ISRCTN 42520374). All study participants provided written informed consent.

### Participants

All At Home/Chez Soi participants met three inclusion criteria: a) ≥ 18 years old; b) absolutely homeless (having no fixed place to stay for at least the past seven nights with little likelihood of finding a place in the upcoming month) or precariously housed (currently occupying a single room in a multi-tenant building or house with shared common areas including bathroom and kitchen [13] or a hotel/motel as a primary residence, and having a history of two or more episodes of absolute homelessness in the past year); and c) presence of a current mental disorder with or without a co-existing substance use disorder, based on the DSM-IV [14] criteria in the Mini International Neuropsychiatric Interview 6.0 (MINI) [15]. Eligible diagnoses included: a) major depressive episode; b) manic or hypomanic episode; c) mood disorder with psychotic features; d) panic disorder; e) posttraumatic stress disorder; or f) psychotic disorder. Diagnosis of a substance use disorder alone did not qualify participants for study entry. Participants were excluded from the study if they: a) were relatively homeless (people who are residing in conditions that do not meet basic standards but who are not absolutely homeless or precariously housed, including those living in overcrowded or hazardous housing, transitional housing such as shelters for domestic abuse, long-term institutions, couch surfing, and people at risk of homelessness or lacking a dwelling for a short period of time due to disasters such as fire or economic situations) [10]; b) lacked a diagnosis of a serious mental disorder; c) had no legal status in Canada; or d) were current recipients of mental health supports via assertive community treatment (ACT) or intensive case management (ICM) services [10].

Participant recruitment was based on referrals from both community social service agencies and acute care services. A targeted recruitment strategy was employed to ensure that the broader study sample adequately represented the target population [16], and all participants were assessed by the intake coordinator who determined study eligibility. Given this study’s focus on black and ethnic minority participants, we recruited extensively among diverse ethnic minority groups through outreach to ethnoracial agencies serving this population.

### Stratification and randomization

Prior to randomization, all eligible participants for the larger trial were stratified into either a high need or moderate need group, indicating their need for mental health services. The stratification algorithm defined high need participants as those who had: 1) community functioning scores from the Multnomah Community Ability Scale (MCAS) ≤ 62 [17]; and 2) a diagnosis of current psychotic or bipolar disorder based on the MINI [15], in addition to meeting one of the following three criteria: i) ≥ 2 hospitalizations for mental illness in any one year in the last 5 years; or ii) diagnosis of comorbid substance use based on the MINI; or iii) recent arrest(s) or incarceration(s) in the past 6 months. High needs participants were randomized to HF with ACT or a usual care group. All other participants were considered to have moderate need for mental health supports services.

Moderate needs participants were further stratified by ethnicity. Self-reported ethnicity was measured using a form validated locally [18], which asked people to select their ethnicity from one of 15 groups based on race and geographical origin: those who selected Black African (e.g. Ghana, Kenya, Somalia), Black Canadian/American, Black Caribbean (e.g. Jamaica, Trinidad, Tobago), East Asian (e.g. China, Japan, Korea), Indian-Caribbean (e.g. Guyana with origins in India), Latin American (e.g. Argentina, Chile, Costa Rica), Middle Eastern (e.g. Egypt, Iran, Israel, Palestine), South Asian (e.g. India, Pakistan, Sri Lanka) and South East Asian (e.g. Malaysia, Philippines, Vietnam) or who reported mixed background that included at least one of the ethnic groups listed above were considered eligible for the adapted HF program. Participants were excluded if they self-identified as Aboriginal, White (European or Canadian) or of a mixed ethnicity that did not include one of the specified groups listed above.

Moderate need participants not belonging to an ethnic minority group were allocated to a regular HF with ICM program or usual care control group. All moderate needs participants belonging to an ethnic minority group (*n* = 237) were randomized to the adapted HF intervention or usual care. A small group of participants (*n* = 33) randomized to the adapted HF program did not receive the adapted intervention either because there was no space available or they requested a non-ethno-racial focused program, and instead received services from the regular HF with ICM program.

Randomization took place via adaptive randomization procedures using a laptop computer connected to the study data management centre: by continuously adjusting the probability of allocation to each treatment group based on existing group assignment, this procedure can produce better balance between treatment groups than strict randomization in small and moderate sized studies [10, 19]. Several aspects of the study prohibited blinding, including the nature of the administered questionnaires (detailed housing history and service use), location of participant interviews (some participants elected to be interviewed at their place of residence) and follow-up procedures (locating participants often required aid from case managers or community workers, where consent was given). As a result, masking follow-up data was also not possible. However, the allocation algorithm was concealed from both the participants and the research staff. Following randomization, participants allocated to the intervention group were immediately connected with their treatment teams, while usual care participants and their referral sources were provided with information about other existing services.

### Interventions

The adapted HF intervention was developed uniquely for the Toronto site with ICM services provided by a mental health agency exclusively serving ethnic minority groups using anti-racist and anti-oppressive frameworks of practice. The model has been described in greater detail elsewhere [11]. The development of the adapted model was informed by practices within the leading agency and a review of the literature [11, 20]. Participants were provided with immediate access to permanent housing of their choice in their preferred neighbourhood (via rent supplements of $600 CAD paid directly to the landlord), in addition to individualized and client-driven mental health support services. Participants worked with a case manager to develop a participant-driven treatment plan, which included both immediate and long-term goals, such as application for disability benefits, access to primary care or other health services in the community, reconnecting with social networks, participation in substance misuse treatment programs and vocational training [21]. The staff ratio in the adapted HF team was 17:1 and services were provided for the duration of the follow-up.

The main principles of anti-racist/anti-oppressive service delivery have been outlined elsewhere [11, 20] and include: empowerment, education, alliance building, language use, alternative healing strategies, advocacy, social justice/activism and fostering reflexivity (critical self-knowledge, awareness and examination of one’s social position and its influence) [20]. HF and anti-racist/anti-oppressive practices share several elements, including a focus on client empowerment and choice, with HF additionally offering targeted strategies to ensure housing stability [11]. The agency offering the adapted HF intervention was committed to anti-racist/anti-oppressive frameworks of practice across program structures and offered regular staff training in such practices, as well as linguistic and culturally accessible programming and services, and a physical environment that is inclusive and welcoming to people from ethnic minority groups [11].

Anti-racist and anti-oppressive staff practices focus on breaking the silence about racism, addressing racism and discrimination, and examining power inequities, oppression and mental health together, taking anti-racist and anti-oppressive action as required [11, 20]. Case managers embrace client-centred, strengths-based, holistic approaches to mental health, recognizing the importance of community of origin, family, and different ways of healing [11, 20]. A key element of the adapted HF program is that case managers were representative of the population they served whenever possible and fluent in the primary language of program participants. In 2009, the agency offered services in 18 languages in addition to English. In addition to ICM services, the agency offered art and music therapy, a community kitchen, computer programs, life skills, traditional Chinese medicine, yoga, English as a second language, as well as support groups for men, women and youth [11, 22]. Another key treatment approach adopted by the agency was the inclusion of families and peer networks early in the recovery process [11, 22].

Individuals randomized to the usual care group were able to access a variety of traditional housing programs, mental health and community services available in the city of Toronto and were provided with information on how to access such services. Toronto is a service rich environment with a variety of health and mental health services, as well as programs specifically serving people experiencing homelessness. Local services include crisis programs, drop-in centres, emergency shelters, inpatient/outpatient mental health services, meal programs, street outreach programs, supportive housing programs, comprehensive primary care teams, ACT and ICM teams [23]. In addition, a variety of specialized primary care and community mental health agencies exist which serve specific ethnic or language groups, immigrants and refugees [23].

### Data collection

Participants were met every 3 months for face-to-face, structured, laptop computer-assisted interviews. Longer interviews took place at baseline, 6-, 12-, 18- and 24-months. During each interview, data was collected and entered wirelessly directly into a secured central database. Brief call-in updates and interviews were conducted with participants on a regular basis to maintain contact and improve follow-up rates. Participants were financially compensated for all interviews and updates ($10 CAD for monthly calls, up to $40 for short interviews and $60 for longer interview). More details on study methodology and design for the At Home/Chez Soi study [10] and on this adapted program, can be found elsewhere [11].

### Outcomes

The prespecified primary study outcome was housing stability, evaluated using the Residential Time Line Follow-Back (RTLFB) Inventory [24]. For each participant, we calculated the percent of days spent in stable housing during the 24-month follow-up period. Stable housing was defined as living either in one’s own apartment, house or room or with family in addition to either tenancy rights or an expectation of residing at this location for ≥ 6 months, as opposed to unstable housing, which included living on the street, temporary residences (< 6 months’ duration and/or no tenancy rights), shelters, crisis units and institutions.

Secondary outcomes explored mental and physical health, social outcomes and services use. Generic quality of life was assessed using the “overall health” Visual Analogue Scale (VAS) of the EuroQol 5 Dimensions (EQ-5D), which measures self-rated health status (both physical and mental) along a scale that ranges from worst (0) to best (100) imaginable health state [25]. Severity of problems with drugs or alcohol within the past month was assessed using the substance use screener (SDScr) of the GAIN Short Screener, GAIN-SS [26]. Additional questions asked about drug and alcohol use related problems and the amount of money spent on alcohol and drugs in the past-month. Psychiatric symptoms within the past month were evaluated using the 14-item modified Colorado Symptom Index (CSI), for which a total score was calculated [27]. Community functioning was evaluated via the total summary score of the MCAS [28]. The total score of the Quality of Life Index (QoLI-20), an instrument used widely in this population, was used for assessing condition-specific quality of life [29]. Physical and psychological community integration were assessed using the separate physical and psychological subscales of the Community Integration Scale (CIS) [30]. Emergency department use was assessed with two questions from the Health Service and Justice Service Use Questionnaire (HSJSU): i) “In the past 6 months, have you been to a hospital emergency room?” and ii) “Approximately how many emergency room visits did you have in total?” The number of days hospitalized was calculated from data collected from the RTLFB by examining the number of overnight stays in hospitals over the study period. The number of police arrests was captured by two additional questions from the HSJSU: i) “In the past six months, have you been arrested?”, and ii) “How many times?”.

### Sample size calculation

The primary outcome of housing stability was used to estimate a clinically meaningful effect size. In a previous randomized trial of supportive housing and ICM compared to standard care among homeless veterans with mental illness and/or substance use [31], participants spent an average of 66 % and 53 % of days housed (of past 90 days) in the intervention and usual care groups, respectively, with an estimated common group standard deviation as 26 %, resulting in a medium effect size (Cohen’s d = 0.5) [32]. In order to account for an assumed attrition rate of 40 %, we estimated that a sample size of 100 participants would be necessary in each arm in our study to provide 80 % power to detect a medium effect size (d = 0.5) [32] at 24 months using a two-sided *t*-test.

### Statistical analyses

Missing data occurred in the key outcomes due to participant withdrawal, non-responses or refusal to answer, which we decided to impute *a priori* using sequential regression multivariate imputation (SRMI) [33]. This method allows for efficient imputation by fitting a model to each variable, conditional on all others, and imputing one variable at a time [34]. Variables included in the multiple imputation model included a) outcome variables collected at all study visits; b) study site; c) age at enrolment; d) gender; e) ethnic minority status and f) Aboriginal status. Imputed values were restricted to the theoretical range of the original variables by use of bounds. Using this approach, 40 imputed datasets were created using the *mi impute chained* command in STATA v.13 (StataCorp LP) and results were combined using PROC MIANALYZE in SAS 9.4 (SAS Institute). Because the extent of missing data was small (5 %), no imputation was performed for percent of days stably housed or the number of hospitalizations, which were calculated for the duration of the follow-up period for each participant for whom housing data was available, and who was known to be alive.

All statistical analyses were performed with SAS version 9.4 on an intention-to-treat basis. The primary outcome (percent of days stably housed during the 24-month follow-up period) and the hospitalization outcome (rate of hospitalization during the 24 months follow-up period) were analysed by fitting mixed models, which assessed the main effect of treatment (adapted HF vs. usual care). For the percent of days stably housed, a linear mixed model was used (PROC MIXED), while a zero-inflated negative binomial (PROC GENMOD) model was fitted for the number of days hospitalized. For all other secondary outcomes for which longitudinal data was available, analyses were conducted using linear mixed models (PROC MIXED) for continuous outcomes and generalized linear models (PROC GENMOD) for count variables. The main effects of time and treatment as well as the interaction of treatment*time were examined. The unstructured covariance matrix for repeated measures was considered in all models. The significance level was set at 5 % for all analyses.

In addition to the outcome analyses, we also examined what baseline variables were associated with the duration of being housed during the study period. In these analyses, the housing outcome was dichotomized into: a) the reference group, which was defined as those who spent less than half of the time (< 50 %) in stable housing during the study period; and b) the group of interest, which was defined as those who had been stably housed half or more of the time (≥ 50 %) during the 24-month study period. These secondary analyses employed a multivariate logistic regression analysis, and were conducted using a two-step process:

1. A list of baseline variables was established, based on potential associations with the outcome variables of interest, and included self-reported demographic variables (age, gender, total length of time homeless, education level, immigrant status, length of residency in Canada, native language), clinical variables (specific MINI diagnoses, including psychosis, major depressive disorder, post-traumatic stress disorder, alcohol or substance abuse or dependence) and the number of self-reported chronic health conditions [10]. Independent logistic models were performed for each baseline variable to test for associations. With the exception of the model examining the treatment variable, all other Step 1 models were adjusted for treatment group (i.e. these were bivariate tests). An *p* < 0.20 was set for these preliminary Step 1 tests, as using an *p* < 0.05 to examine potential confounders can lead to deletion of important confounder variables from the model [35].
2. All covariates that were identified as significant in Step 1 at *p* < 0.20 were added into a multivariate logistic regression model. If two or more of the selected variables from Step 1 were highly correlated (e.g. age and length of time in Canada), we performed separate models with each of these variables, but only retrained one of the highly correlated variables in the multivariate model based on strength of effect in the model.

## Results

**Fig. 1:**
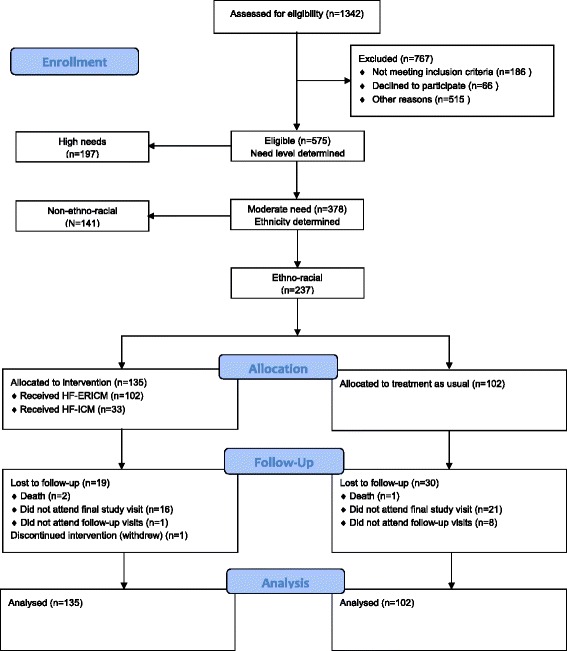
Consort diagram showing participant flow through study

Between October 28, 2009 and June 29, 2011, a total of 237 participants were recruited and randomized to either the adapted HF program (*n* = 135) or usual care (*n* = 102) (Fig. 1). Follow-up interviews were completed for 90 % (*n* = 213) of participants at 12 months and 79 % (*n* = 188) of participants at 24 months. By study end, 49 participants were lost to follow-up and one withdrew.

Baseline and demographic characteristics were similar between the groups (Table 1). Participants were mostly single and never married (69 %), male (67 %) and had been homeless for an average of 3.9 years SD 5.0 (median 2.1). While nearly half were native English speakers (48 %), more than two-thirds were born outside of Canada (72 %). The most common ethnic groups reported were Black-Caribbean (22 %), Black-African (18 %), Black-Canadian (14 %), Mixed (11 %) and South Asian (10 %). Based on the MINI, the most common diagnoses in our sample were depressive disorder (41 %), psychotic disorder (36 %), post-traumatic stress disorder (24 %) and mood-disorder with psychotic features (23 %). Substance use comorbidities were common, with more than one-third (34 %) meeting criteria for alcohol abuse or dependence and 36 % meeting criteria for substance abuse or dependence. Suicidality was common, with nearly one-third of our sample (29 %) reporting moderate to high suicidality.

**Table 1:** Participant demographics at study baseline^a^

Characteristic	Adapted Housing First (*n* = 135)	Usual care (*n* = 102)
Age, *n* (%)		
< 30	40 (29.6)	28 (27.5)
30–39	33 (24.4)	21 (20.6)
40–49	38 (28.1)	35 (34.3)
≥ 50	24 (17.8)	18 (17.6)
Male, *n* (%)^b^	91 (67.9)	65 (66.3)
Country of birth, *n* (%)		
Canada	37 (27.4)	29 (28.4)
Other	98 (72.6)	73 (71.6)
Native language, *n* (%)		
English	65 (48.1)	48 (47.1)
Other	70 (51.9)	54 (52.9)
Ethnicity, *n* (%)		
Black - Caribbean Region	32 (23.7)	21 (20.6)
Black - Africa	25 (18.5)	17 (16.7)
Black - Canada	21 (15.6)	13 (12.7)
Mixed background^c^	9 (6.7)	17 (16.7)
South Asian	13 (9.6)	10 (9.8)
Other^d^	35 (25.9)	24 (23.5)
Marital status *n* (%)		
Single, never married	89 (66.4)	73 (71.6)
Other^e^	45 (33.6)	29 (28.4)
Number of children, *n* (%)		
0	95 (70.4)	74 (72.5)
1	24 (17.8)	13 (12.7)
≥ 2	16 (11.8)	15 (14.7)
Current housing status, *n* (%)		
Absolutely homeless	121 (89.6)	93 (91.2)
Precariously housed	14 (10.4)	9 (8.8)
Total length of homelessness, years, mean ± sd^f^	3.5 ± 4.4	4.4 ± 5.7
Longest period of homelessness, years, mean ± sd^g^	1.9 ± 3.0	2.2 ± 2.8
Education history, *n* (%)		
< High school	61 (45.2)	45 (44.1)
Completed high school	22 (16.3)	23 (22.5)
Some post-secondary school	52 (38.5)	34 (33.3)
MCAS total score, mean ± sd	65.5 ± 3.12	65.4 ± 3.25
MINI results, *n* (%)^h^		
Depressive episode	57 (42.2)	39 (38.2)
Manic or hypomanic episode	12 (8.9)	7 (6.9)
Post-traumatic stress disorder	34 (25.2)	23 (22.5)
Panic disorder	21 (15.6)	18 (17.6)
Mood disorder with psychotic features	30 (22.2)	24 (23.5)
Psychotic disorder	46 (34.1)	40 (39.2)
Alcohol abuse or dependence	43 (31.9)	38 (37.3)
Substance abuse or dependence	45 (33.3)	41 (40.2)
Suicidality^i^	36 (26.7)	32 (31.4)

*MCAS* Multnomah Community Ability Scale, *MINI* Mini International Neuropsychiatric Interview

^a^The following variables had missing values: marital status (*n* = 1); total length of homelessness (*n* = 5); longest period of homelessness (*n* = 1). Percentages were calculated out of the total available data

^b^Individuals who self-identified as female and other/transgendered/transsexual are not listed due to small cell size ≤ 5 for the latter category

^c^“Mixed” ethnicity included individuals who had at least one parent from the following ethnicities: East Asian, South East Asian, African Black, Canadian Black, Caribbean Black, Latin American, Indian-Caribbean and Middle Eastern

^d^The “Other” category comprised of individuals who self-identified to one of the following groups but were suppressed due to small cell size: East Asian, Indian-Caribbean, Latin American, Middle Eastern and South East Asian

^e^“Other” marital status categories include the following options: married, cohabitating with partner, divorced, separated, widowed

^f^Median values in years for total length of homelessness were as follows: 2.0 for the adapted HF group and 2.5 for the usual care group

^g^Median values in years for longest period of homelessness were as follows: 1.0 for the adapted HF group and 1.0 for the usual care group

^h^MINI Diagnoses all represent current diagnoses established at baseline

^i^Suicidality was assessed as low, medium, or high; results here are shown for the collapsed moderate and high categories

During the 24-month study period, participants randomized to the adapted HF group were housed 75 % (95 % CI 70-81) of the time, compared to usual care participants, who were housed 41 % (95 % CI 35-48) of the time (difference = 34 %, 95 % CI 25 to 43). Results from a zero-inflated negative binomial model found that the number of self-reported days spent in hospital did not differ significantly between the treatment allocation groups (rate ratio = 1.9, 95 % CI 0.78 to 4.5).

Comparing 24 month values to baseline for secondary outcomes (Table 2), we only observed a significant treatment*time interaction for community functioning (MCAS), with greater improvements among participants in the adapted HF group, compared to those in the usual care group (change in mean difference = 2.2, 95 % CI 0.06 to 4.3). Although generic quality of life (EQ5D VAS) (change in mean difference = 6.9, 95 % CI 0.17 to 14.0), severity of substance use problems (GAIN) (ratio of rate ratios = 0.62, 95 % CI 0.39 to 0.98) and the number of days experiencing problems due to alcohol (ratio of rate ratios = 0.35, 95 % CI 0.14 to 0.88) improved significantly from baseline to 12 months among adapted HF compared to usual care participants; these treatment* time interactions were no longer statistically significant at 24 months.

**Table 2:** Treatment group changes from baseline^a^

Outcome	12 months	*P*	24 months	*P*
Health status (EQ5D-VAS)	6.91 (0.17 to 13.66)	0.045	−0.12 (-7.09 to 6.84)	0.97
Mental illness symptomatology (CSI)	−0.22 (-3.42 to 2.98)	0.89	0.04 (-3.39 to 3.46)	0.98
Substance use problem severity (GAIN-SS)	0.62 (0.39 to 0.98)	0.04	1.00 (0.61 to 1.64)	> 0.99
Physical community integration (CIS-PHYS)	0.98 (0.79 to 1.21)	0.84	0.86 (0.70 to 1.07)	0.17
Psychological community integration (CIS-PSYCH)	0.27 (-0.96 to 1.49)	0.67	0.61 (-0.64 to 1.86)	0.34
Community functioning (MCAS)	1.25 (-0.72 to 3.21)	0.21	2.16 (0.06 to 4.26)	0.04
Quality of life (QoLI)	1.83 (-4.70 to 8.36)	0.58	2.94 (-3.55 to 9.42)	0.37
Number of emergency department visits	0.74 (0.36 to 1.51)	0.41	0.67 (0.28 to 1.58)	0.36
Number of arrests	1.66 (0.51 to 5.39)	0.40	1.31 (0.37 to 4.62)	0.67
Amount of money spent on alcohol in past 30 days	−30.76 (-80.58 to 19.06)	0.23	−9.99 (-47.62 to 27.64)	0.60
Amount of money spent on drugs (not prescription) in past 30 days	15.41 (-132.47 to 163.29)	0.84	84.32 (-35.09 to 203.74)	0.17
Number of days in past 30 experienced alcohol problems	0.35 (0.14 to 0.88)	0.03	0.35 (0.12 to 1.02)	0.054
Number of days in the past 30 experienced drug problems	0.73 (0.34 to 1.57)	0.43	0.58 (0.24 to 1.42)	0.23

^a^The change from baseline to the other study time points corresponds to the mean change (95 % CI) for continuous outcomes and the ratio of the rate ratios (95 % CI) for count outcomes. *P* values were assessed on the basis of the treatment group*time interaction. For continuous outcomes, the time by treatment group interaction examined the change in the mean from baseline to a subsequent follow-up visit (12- and 24-months) for the adapted HF group compared to the usual care group and 95 % CI. For count outcomes (substance use problem severity, physical community integration, number of emergency department visits, number of arrests, days experiencing problems due to alcohol, days experiencing problems due to drugs), the treatment group*time interaction evaluated the ratio of rate ratios for each post-baseline visit (e.g. rate ratio at follow-up visit relative to baseline in the adapted HF group divided by the rate ratio at follow-up visit relative to baseline in the usual care group) and 95 % CI. In these analyses, baseline values were used as a reference time point for all comparisons at subsequent time points (12- and 24-months) and the usual care group was used as a reference group

Additional file 1: Table S1 shows model-estimated outcome values at each study time point, by treat ment group.

The results of the unadjusted and adjusted logistic regressions for the secondary data analyses are found in Table 3. In total, 65 % of participants were stably housed for 50 % of the time or more. In preliminary bivariate logistic regression analyses (adjusting for treatment group) with *p* < 0.20 (Table 3, left column), male gender and MINI diagnosis of psychotic disorder were associated with reduced odds of being housed 50 % of the time, while diagnosis of major depression and post-traumatic stress disorder were both associated with increased odds of being housed 50 % of the time or more. These four variables were then included into the multivariate model, in addition to the treatment group.

**Table 3:** Odds ratios from unadjusted and adjusted logistic regression models examining the effect of baseline measures on percent of time spent stably housed during 24 months of study (< 50 % of time vs. ≥ 50 % of time)

Baseline variables	Unadjusted^a^		Adjusted	
	OR (95 % CI)	*P*	OR (95 % CI)	*P*
Treatment group	7.17 (3.83 to 13.42)	< 0.001	7.86 (4.06 to 15.23)	< 0.001
Age (years)	1.01 (0.98 to 1.04)	0.47		
Gender	0.57 (0.29 to 1.12)	0.10	0.58 (0.29 to 1.16)	0.12
Total length of homelessness (years)	0.96 (0.91 to 1.02)	0.24	-	-
High School or higher education	0.73 (0.39 to 1.37)	0.33	-	-
Immigrant Status	0.92 (0.46 to 1.84)	0.82	-	-
Length of time in Canada	0.99 (0.96 to 1.02)	0.36	-	-
English was first language	1.07 (0.57 to 1.99)	0.83	-	-
MINI diagnoses				
Psychosis	0.34 (0.17 to 0.65)	0.001	0.37 (0.18 to 0.75)	0.01
Major depressive episode	1.55 (0.81 to 2.96)	0.18	1.03 (0.50 to 2.10)	0.95
Alcohol or substance abuse or dependence	1.10 (0.59 to 2.05)	0.77	-	-
Post-traumatic stress disorder	1.75 (0.80 to 3.84)	0.16	1.32 (0.58 to 3.05)	0.51
Number of chronic medical conditions	1.06 (0.95 to 1.18)	0.28	-	-

^a^All univariate analyses were adjusted for treatment groups (adapted HF vs. usual care). Reference categories for categorical variables were as follows (indicated by 0): treatment group (usual care = 0, adapted HF = 1); gender (female/other = 0, male = 1); education (less than high school = 0; completed high school/some college/university = 1); immigrant status (Canadian born = 0; foreign born = 1); English as first language (other languages = 0; English = 1); MINI diagnoses (absence of diagnosis = 0; presence of diagnosis = 1)

In the final adjusted multivariate logistic model, with *p* < 0.05 (Table 3, right column), participants allocated to the treatment group were almost eight times more likely to be housed 50 % or more of the time over the 24-month study period (OR = 7.9, 95 % CI 4.1 to 15.2) compared to those in the usual care group. Compared to those without a baseline diagnosis of psychosis, participants with this diagnosis had a reduced likelihood of being housed 50 % or more of the time during the study period (OR = 0.37, 95 % CI 0.18 to 0.72). Neither gender (OR = 0.58, 95 % CI 0.29 to 1.6), post-traumatic stress disorder (OR = 1.3, 95 % CI 0.58 to 3.1), nor major depression (OR = 1.0, 95 % CI 0.50 to 2.1) showed a statistically significant association with housing in adjusted analyses.

## Discussion

Despite documented health disparities among people from black and ethnic minority groups, the literature on interventions aiming to improve health and social outcomes for this disadvantaged population is scant. People from black and ethnic minority groups experiencing homelessness and mental illness face additional challenges, as traditional housing and support strategies may not adequately address their needs. To our knowledge, this is the first study examining the effectiveness of a HF program which has been specifically adapted for this population. Enhancing HF with anti-racist/anti-oppressive principles of practice for black and ethnic minority homeless adults with mental illness successfully improved housing stability and community functioning for this population, compared to usual care, at the Toronto site of the At Home/Chez Soi trial. Our findings are consistent with previous studies of HF using rent supplements and mental health services (ACT or ICM) which report improvements in housing stability [8, 31, 36], among homeless participants with mental illness. This study adds to this literature base by demonstrating the effectiveness of a HF adaptation, using anti-racist/anti-oppressive practice, in improving housing stability among homeless adults with mental illness from ethno-racial minority groups, a population that may be reluctant to engage with traditional services and is at risk of poor outcomes [1, 2, 5].

Interestingly, secondary analyses showed that diagnosis of psychosis was the only baseline variable examined found to be associated with housing stability over time. A previous single-site HF intervention that provided services via ICM found that psychotic symptom severity was similarly associated with leaving the project [37]. It is possible that the service intensity offered by ICM-based HF programs is not sufficient for some individuals experiencing psychotic symptoms, and that more intensive support services such as ACT may be needed for these participants.

We also observed that the adapted HF model led to improvements in community functioning among participants. Community functioning is a complex concept that encompasses symptom interference with functioning, adjustment to living, social competence and behavioural problems [17]. As part of the Community Mental Health Evaluation Initiative (CMHEI), participants with severe mental illness from three Canadian cities (Hamilton, Ottawa, Toronto) who were either homeless or at risk of homelessness were provided with various types of housing and supports (*n* = 91) and saw a significant improvement in MCAS scores from study baseline to 9-months of a magnitude of three points [38]. In the present study, treatment participants improved approximately four points from baseline, which was indicative of a small effect size (Cohen’s d = 0.27) [17, 39].

Unlike housing outcomes, substance use related outcomes have not shown consistent improvements among participants in HF programs. Reductions in alcohol use have been reported by a previous study examining a single-site HF intervention for adults with severe alcohol use disorders [40], but others, serving different subpopulations of homeless people, have found no changes in either drug or alcohol use after program enrolment [31]. The rates of alcohol or substance abuse or dependence (46 %) at study entry in our sample were comparable to a previous randomized trial of supported housing which also failed to see improvement in substance use problems (50 %) [31].

Previous reviews have confirmed that culturally adapted behavioural interventions result in better health outcomes compared to usual care or other controls [41]; however, few culturally adapted programs have been evaluated for superiority over the intervention they were adapted from, both because there is a lack of willingness to provide unaltered programs for cultural groups with known preferences (e.g. for language) but also due to the costs such comparisons would accrue [41]. The adapted HF program described here combines HF principles with anti-racist and anti-oppressive practice. The availability of this approach, in addition to more traditional HF programs, may offer ethno-racial minority participants a choice of mental health support services that may be more closely aligned with their personal preferences and support needs. Addressing experiences of racism and oppression may be an important factor in engaging in treatment and building a working alliance with some participants, which are important components of successful treatment outcomes [42]. Furthermore, increasing treatment choice may also lead to improved outcomes: a previous study of participants in a Housing First program reported that increased perceived choice is strongly inversely associated with psychiatric symptoms [43].

We have established the effectiveness of a HF program adapted for homeless adults with mental illness from black and ethnic minority groups compared to usual care. Future research should compare housing outcomes in traditional and adapted HF interventions to identify key ingredients of program success. As choice and plurality of services are important elements of healthcare in Canada, this study aimed to examine the effectiveness of the adapted HF intervention compared to usual services in the community. Our results therefore demonstrate the real world benefit of adapted HF in the Canadian context, supporting its inclusion in the complement of services and supports targeting black and ethnic minority groups.

This study has some additional limitations. Firstly, the ethnic diversity in Toronto is more extensive compared to most large urban centres which therefore may not face similar challenges. Secondly, we have shown that the intervention works among this specific sample of individuals of ethnoracial ethnicity residing in Toronto, Canada. However, the unique ethno-cultural composition of our sample and the services available to the residents of Toronto may limit the generalizability of our findings to other populations of homeless adults with mental illness from different ethnic backgrounds and/or in other service delivery contexts. Third, this study uses self-reported data and therefore may be limited by recall bias and social desirability bias. Fourth, an economic evaluation has not yet been conducted, but will be the focus of future work. Fifth, while our targeted recruitment strategy attempted to capture a representative sample of homeless adults with mental illness from ethnic minority groups in Toronto, we cannot confirm that we succeeded in this goal due to the lack of accurate data on this difficult to reach population.

A key strength of this study is its ability to maintain high follow-up rates among a difficult to track population, which was greatly helped by monthly checks with participants and acquiring consent to contact service providers and family/friends for current participant contact information. More details on other strategies employed by study staff are described elsewhere [10, 12].

Our findings add to the literature on cultural adaptations for groups experiencing severe disadvantage. Future studies comparing anti-racist/anti-oppressive adaptations to usual HF services and eliciting client preferences of and satisfaction with adapted services may better inform planning and resource allocation.

## Conclusions

A Housing First intervention adapted using anti-racist and anti-oppression principles improved housing and community functioning outcomes in a diverse group of homeless ethnic minority participants with mental illness compared to usual care. The culturally adapted intervention may increase access to care through offering choice to racialized populations.

## Additional file

Additional file 1: Table S1. Estimated outcome values at each study visit, by treatment group. (DOCX 13.9 kb)

## Abbreviations

ACT: Assertive community treatment
AO: Anti-oppression
AR: Anti-racism
HF: Housing First
ICM: Intensive case management
MCAS: Multnomah community ability scale
MINI: Mini international neuropsychiatric interview
