# Supplementary material for: The effectiveness of a Housing First adaptation for ethnic minority groups: findings of a pragmatic randomized controlled trial
Source: BMC Public Health. 2016 Oct 21;16:1110. doi: 10.1186/s12889-016-3768-4 (PMC5073863; doi:10.1186/s12889-016-3768-4)
Supplement: Additional file 1: Table S1. — Estimated outcome values at each study visit, by treatment group. (DOCX 13.9 kb) [file 12889_2016_3768_MOESM1_ESM.docx]

**Additional file 1: Table S1.** Estimated outcome values at each study visit, by treatment group.^1^

| Outcome | Baseline | | 6 Months | | 12 Months | | 18 Months | | 24 Months | |
| --- | --- | --- | --- | --- | --- | --- | --- | --- | --- | --- |
|  | Adapted HF | TAU | Adapted HF | TAU | Adapted HF | TAU | Adapted HF | TAU | Adapted HF | TAU |
| Health Status (EQ5D-VAS) | 60.57 (56.48 to 64.66) | 65.55 (60.78 to 70.31 | 65.70 (61.35 to 70.04) | 66.60 (61.22 to 71.99 | 67.06 (62.96 to 71.17) | 65.12 (60.05 to 70.20) | 65.32 (61.14 to 69.50) | 71.78 (66.64 to 76.92) | 67.01 (63.08 to 70.93) | 72.11 (67.42 to 76.79) |
| Mental Illness Symptomatology (CSI) | 39.97 (37.91 to 42.02) | 40.83 (38.46 to 43.20 | 34.75 (32.65 to 36.86) | 36.94 (34.51 to 39.37 | 33.23 (31.17 to 35.29) | 34.31 (31.79 to 36.84) | 31.94 (29.96 to 33.92) | 32.37 (30.03 to 34.71) | 31.91 (29.90 to 33.92) | 32.73 (30.29 to 35.17) |
| Substance Use Problem Severity (GAIN-SS) | 1.16 (0.87 to 1.46) | 1.27 (0.92 to 1.63 | 1.10 (0.82 to 1.38) | 1.04 (0.70 to 1.38 | 0.72 (0.48 to 0.97) | 1.28 (0.90 to 1.66) | 0.81 (0.57 to 1.05) | 0.94 (0.63 to 1.25) | 0.74 (0.51 to 0.97) | 0.81 (0.52 to 1.10) |
| Physical Community Integration (CIS-PHYS) | 2.41 (2.10 to 2.71) | 2.48 (2.18 to 2.77 | 2.31 (1.95 to 2.67) | 2.29 (1.91 to 2.67 | 2.47 (2.12 to 2.81) | 2.60 (2.19 to 3.00) | 2.37 (2.04 to 2.70) | 2.70 (2.29 to 3.11) | 2.32 (1.98 to 2.67) | 2.77 (2.38 to 3.16) |
| Psychological Community Integration (CIS-PSYCH) | 10.86 (10.23 to 11.50) | 11.12 (10.38 to 11.85 | 12.05 (11.42 to 12.68) | 11.30 (10.54 to 12.05 | 12.34 (11.69 to 12.99) | 12.33 (11.51 to 13.15) | 12.33 (11.73 to 12.92) | 12.79 (11.96 to 13.62) | 12.99 (12.37 to 13.61) | 12.63 (11.90 to 13.37) |
| Community Functioning (MCAS) | 65.63 (65.08 to 66.19) | 65.44 (64.79 to 66.08 | 67.48 (66.37 to 68.59) | 65.48 (64.15 to 66.82 | 67.18 (65.95 to 68.41) | 65.74 (64.11 to 67.36) | 68.89 (67.53 to 70.25) | 67.22 (65.52 to 68.92) | 69.78 (68.41 to 71.14) | 67.42 (65.75 to 69.09) |
| Quality of Life (QoLI) | 71.67 (67.82 to 75.52) | 73.31 (68.88 to 77.75 | 83.81 (80.12 to 87.50) | 79.16 (74.95 to 83.38 | 86.17 (82.27 to 90.08) | 85.99 (81.27 to 90.71) | 85.84 (82.02 to 89.66) | 83.98 (79.43 to 88.53) | 89.09 (85.23 to 92.96) | 87.80 (83.20 to 92.40) |
| Number of Emergency Department Visits | 1.24 (0.76 to 1.71) | 0.83 (0.61 to 1.06 | 0.61 (0.34 to 0.89) | 0.83 (0.23 to 1.43 | 0.75 (0.50 to 1.00) | 0.69 (0.36 to 1.02) | 0.74 (0.44 to 1.04) | 0.45 (0.02 to 0.88) | 0.58 (0.41 to 0.75) | 0.60 (0.10 to 1.10) |
| Number of arrests | 0.29 (0.18 to 0.39) | 0.62 (0.13 to 1.12 | 0.14 (0.07 to 0.20) | 0.14 (-0.01 to 0.30 | 0.15 (0.07 to 0.23) | 0.20 (0.03 to 0.37) | 0.09 (0.03 to 0.15) | 0.15 (0.06 to 0.24) | 0.10 (0.03 to 0.18) | 0.17 (0.06 to 0.29) |
| Amount of money spent on alcohol in past 30 days | 32.30 (14.58 to 50.01) | 36.50 (15.79 to 57.20 | 31.34 (9.01 to 53.67) | 46.18 (13.73 to 78.63 | 27.68 (2.83 to 52.53) | 62.63 (22.28 to 102.99) | 28.05 (12.65 to 43.44) | 41.36 (19.17 to 63.56) | 30.40 (12.38 to 48.42) | 44.59 (18.72 to 70.45) |
| Amount of money spent on drugs (not prescription) in past 30 days | 78.43 (-0.71 to 157.56) | 180.94 (89.07 to 272.80 | 61.33 (-3.80 to 126.46) | 104.91 (4.12 to 205.71 | 78.63 (0.03 to 157.23) | 165.73 (65.09 to 266.38) | 49.41 (6.09 to 92.74) | 83.47 (25.88 to 141.06) | 54.31 (7.84 to 100.77) | 72.49 (21.97 to 123.01) |
| Number of days in past 30 experienced alcohol problems | 3.37 (1.94 to 4.79) | 2.29 (0.96 to 3.62 | 2.63 (1.27 to 3.99) | 3.41 (1.53 to 5.29 | 1.41 (0.40 to 2.43) | 2.75 (0.97 to 4.52) | 1.63 (0.54 to 2.72) | 2.51 (0.86 to 4.17) | 1.12 (0.30 to 1.93) | 2.17 (0.73 to 3.61) |
| Number of days in the past 30 experienced drug problems | 4.40 (2.81 to 5.99) | 4.47 (2.57 to 6.37 | 2.35 (1.00 to 3.69) | 4.18 (2.11 to 6.25 | 2.72 (1.31 to 4.12) | 3.77 (1.80 to 5.74) | 2.46 (1.10 to 3.81) | 2.28 (0.60 to 3.96) | 1.88 (0.71 to 3.06) | 3.29 (1.43 to 5.16) |

^1^ Estimated means (95% CI) for continuous outcomes and rate ratios (95% CI) for count outcomes were generated from models of outcome measures assessing the effect of time and treatment and the treatment x time interaction. Count outcomes were limited to: substance use problem severity, physical community integration, number of emergency department visits, number of arrests, days experiencing problems due to alcohol, days experiencing problems due to drugs.
